# Simplified Machine Learning Models Can Accurately Identify High-Need High-Cost Patients With Inflammatory Bowel Disease

**DOI:** 10.14309/ctg.0000000000000507

**Published:** 2022-06-07

**Authors:** Nghia H. Nguyen, Sagar Patel, Jason Gabunilas, Alexander S. Qian, Alan Cecil, Vipul Jairath, William J. Sandborn, Lucila Ohno-Machado, Peter L. Chen, Siddharth Singh

**Affiliations:** 1 Division of Gastroenterology, Department of Medicine, University of California San, Diego, La Jolla, California, USA;; 2 Division of Gastroenterology, Department of Medicine, USA;; 3 Department of Epidemiology and Biostatistics, Western University, London, Ontario, Canada;; 4 Division of Biomedical Informatics, Department of Medicine, University of California San Diego, La Jolla, California, USA.

## Abstract

**INTRODUCTION:**

Hospitalization is the primary driver of inflammatory bowel disease (IBD)-related healthcare costs and morbidity. Traditional prediction models have poor performance at identifying patients at highest risk of unplanned healthcare utilization. Identification of patients who are high-need and high-cost (HNHC) could reduce unplanned healthcare utilization and healthcare costs.

**METHODS:**

We conducted a retrospective cohort study in adult patients hospitalized with IBD using the Nationwide Readmissions Database (model derivation in the 2013 Nationwide Readmission Database and validation in the 2017 Nationwide Readmission Database). We built 2 tree-based algorithms (decision tree classifier and decision tree using gradient boosting framework [XGBoost]) and compared traditional logistic regression to identify patients at risk for becoming HNHC (patients in the highest decile of total days spent in hospital in a calendar year).

**RESULTS:**

Of 47,402 adult patients hospitalized with IBD, we identified 4,717 HNHC patients. The decision tree classifier model (length of stay, Charlson Comorbidity Index, procedure, Frailty Risk Score, and age) had a mean area under the receiver operating characteristic curve (AUC) of 0.78 ± 0.01 in the derivation data set and 0.78 ± 0.02 in the validation data set. XGBoost (length of stay, procedure, chronic pain, drug abuse, and diabetic complication) had a mean AUC of 0.79 ± 0.01 and 0.75 ± 0.02 in the derivation and validation data sets, respectively, compared with AUC 0.55 ± 0.01 and 0.56 ± 0.01 with traditional logistic regression (peptic ulcer disease, paresthesia, admission for osteomyelitis, renal failure, and lymphoma) in derivation and validation data sets, respectively.

**DISCUSSION:**

In hospitalized patients with IBD, simplified tree-based machine learning algorithms using administrative claims data can accurately predict patients at risk of progressing to HNHC.

## Graphical abstract

**Figure:**
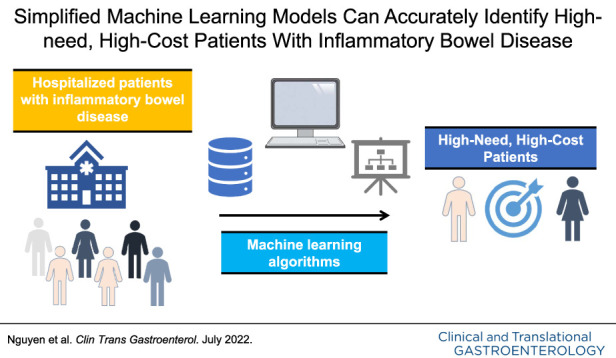

## INTRODUCTION

Inflammatory bowel disease (IBD) is a chronic, high-cost condition that affects more than 1.6–3.1 million people in the United States and with annual costs exceeding $25.6 billion (1,2). Although pharmaceutical costs are increasing, the primary driver of IBD-related healthcare costs and morbidity continues to be unplanned healthcare utilization, with hospitalization and emergency department visits accounting for 56% of total healthcare costs in the United States (3). Approximately 22%–45% patients with IBD are hospitalized within 5 years of diagnosis; 1 in 5 hospitalized patients with IBD is readmitted within 30 days (4).

Previous studies in healthcare policy have identified a subset of high-need, high-cost (HNHC) patients with complex chronic medical conditions who account for a significant proportion of healthcare spending and experience poor quality in their care (5–7). In a nationally representative longitudinal cohort study, using the 2013 Nationwide Readmission Database (NRD) , we observed that hospitalized patients with IBD spend a median of 6 days in the hospital annually, with a subset of HNHC patients spending over 45 days in the hospital annually, with 1 hospitalization every 2 months, and accounted for 38% of total hospitalization costs (with median annual hospitalization costs ∼$90,000) in patients with IBD (8). With escalating costs of IBD care, population health management strategies are needed to promote value-based care in IBD (9). Accurate identification of HNHC patients is the critical first step for population health management. Burden and drivers of healthcare utilization are distinctly different in HNHC patients, and personalized interventions targeting these patients may be highly effective in decreasing healthcare costs. However, current regression-based models to identify hospitalized patients who may progress to HNHC status have modest discriminative performance (4). This may be due to failure to recognize and account for a large number of potential risk factors and inability to account for nonlinear relationships. Data-driven machine learning (ML) models may overcome these limitations and accurately inform likelihood of progression to HNHC status (10). Waljee et al. (11) have previously demonstrated that a ML approach could accurately predict the combined end point of initial hospitalization and/or corticosteroid use within 6 months of an IBD diagnosis in 20,368 patients in the Veterans Health Administration. However, no ML algorithms have been developed to identify hospitalized patients at high risk of readmission and progressing to high burden of unplanned healthcare utilization.

To accurately identify hospitalized patients with IBD likely to progress to HNHC, we sought to develop and validate novel, prognostic ML algorithms in a nationally representative longitudinal cohort of 47,402 hospitalized adult patients with IBD, using NRD 2013 and externally validate our findings in NRD 2017. We compared the predictive performance of the ML model with a traditional logistic regression (LR) model.

## METHODS

### Data sources and derivation/validation cohorts

For our derivation cohort, we used a previous cohort from NRD 2013 (8). Briefly, NRD is a nationally representative longitudinal database that tracks hospitalized patients from 21 state inpatient databases throughout the country in 1 calendar year and accounts for 49.3% of the US population. For our external validation cohort, we used NRD 2017. Because the NRD is a publicly available database that contains only deidentified patient information, this study was deemed exempt from Institutional Review Board.

### Study population

We included adults (age ≥18 years) admitted with a primary or secondary discharge diagnosis of IBD between January and June 2013, followed for subsequent hospitalization until December 2013 or death. We used the Clinical Classifications Software (CCS) for *International Classification of Diseases, Clinical Modification*-9 with CCS code 144 to identify patients with IBD. Details of this cohort are described elsewhere (8). After the first admission with a discharge diagnosis of IBD, patients were deemed to be ‟at risk” for hospitalization and contributed to follow-up time till December 31, 2013, or death (see Supplementary Figure 1, http://links.lww.com/CTG/A827). For our external validation cohort, we relied on *International Classification of Diseases, Clinical Modification*-10 codes (K50.x for Crohn's disease and K51.x for ulcerative colitis) to identify patients with IBD because there were no CCS codes available in NRD 2017.

We excluded patients with the following criteria: (i) age younger than 18 years at the time of index hospitalization, (ii) index hospitalization between July and December 2013, (iii) transferred from another hospital, (iv) missing data for length of hospital stay, or (v) missing data on hospital charges for a given admission.

### Outcomes measured

Our primary outcome was identifying hospitalized patients with IBD at highest risk of ongoing unplanned healthcare utilization and likelihood of becoming HNHC over the course of a year, based on ‟total days spent in hospital per year.” Patients in the top decile based on total days spent in the hospital per year were considered HNHC (8). Our secondary outcome was risk of 90-day readmission after index hospitalization.

### Predictor variables

Independent predictor variables included patient, hospitalization-related, and hospital factors that have previously been identified in NRD and other databases. These features were generated from previous work using the same NRD 2013 database such as the Hospital Frailty Risk Score (an independent predictor of serious infections and hospitalizations in patients with IBD), Charlson Comorbidity Index (measurement of comorbid medical conditions), and IBD-related procedures (12–14). Variables included in our models were collected at the time of the index hospitalization. In total, we considered 107 different features for further analysis, and after accounting for missing values, our data set included a total of 46,586 patients. We calculated a correlation matrix on the entire data set and removed correlated features based on Pearson correlation >0.75, leaving us with 101 features to build our models.

### Statistical analysis

We used descriptive statistics to compare HNHC vs non-HNHC patients with IBD at the index hospitalization. We performed the Pearson χ^2^ test and Student *t* test to compare parametric categorical and continuous variables, respectively. For nonparametric categorical and continuous variables, we performed Fisher exact and Wilcoxon rank sum tests, respectively. All hypothesis testing were performed with a 2-sided *P* value with a statistical significance threshold of <0.05. We performed all statistical analyses with Stata MP (2015, Stata Statistical Software: Release 14; StataCorp, College Station, TX).

#### Development of prediction models

**Figure 1.**
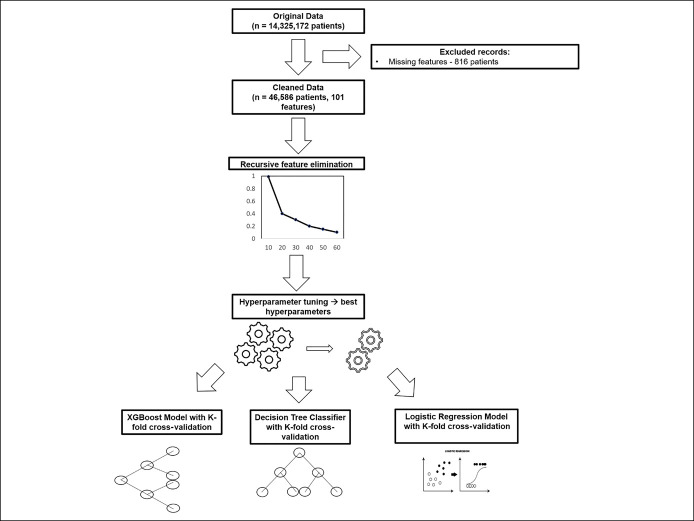
Workflow diagram for model development.

Model building was performed in accordance with the recommendations as outlined by PROBAST (15). We chose a decision tree classifier (DTC) with random forest-based models as our supervised ML algorithms because they are intuitive, easy to interpret, and easy to adopt. Random forest is an ensemble ML approach that uses a collection of decision trees to reduce bias and variance in classifying observations. When all the votes from all the trees are combined, the most popular vote is considered the final predicted outcome. We developed a second decision tree-based algorithm using gradient boosting (XGBoost), which is a method of sequentially building individual decision trees with each new tree helping to correct errors made by a previously trained tree. We used grid search hyperparameter optimization to identify the best hyperparameters for the model. To compare the performance of our ML models, we also developed a traditional LR model. A flow diagram for the development of our models is described in Figure 1. For our models, we estimated out-of-sample performance of our models and accounted for potential overfitting with *k*-fold cross-validation (with 10 folds) to internally validate the findings of our model. Model building and feature selection, using recursive feature elimination (RFE), were performed using the scikit-learn package in the Python programming language. Visualizations were produced using the seaborn package with scikit-learn (16,17). Hyperparameter optimization was tuned using the GridSearch function in scikit-learn, and the best hyperparameters were applied to both tree-based and LR models (16,17).

#### Simplified tree-based algorithms and variable importance plots

To develop simplified models with high performance, we used RFEwith a random forest model trained on the entire data set (16). RFE is a feature importance selection method that computes an importance score for a model built on all features and removes the weakest feature (or features) until the specified number of features is reached. For our simplified models, we set a specified limit of 5 features for the final model and secondary analyses with 10-feature models for both our primary and secondary outcomes. For our primary outcome, we also developed models while excluding the variable “length of stay (LOS)” at the index hospitalization. To compare the performance of our models, we evaluated discrimination as measured by receiver operating characteristics area under the curve (AUC), recall and precision at a threshold of 0.50, and accuracy with *k*-fold cross-validation with 10 as the default number of folds. Mean AUC and accompanying SD are presented in the figures. We developed a workflow diagram (Figure 1) to outline the steps that were undertaken to develop our models.

#### Code repository

Our code to perform the analysis in Python is available in a public GitHub repository at https://github.com/Autonomousse/UCSD_NRD_2017.

## RESULTS

### Patient demographics

Of a total of 14,325,172 discharge records included in the NRD 2013, we identified 94,498 records that were potentially eligible for analysis. A total of 47,402 adult patients with IBD with index hospitalizations between January and June 2013 were identified, and 46,586 patients with complete data were ultimately included in the final analysis (Figure 1). A full description of our cohort is described elsewhere (12). Compared with non-HNHC patients, those who were HNHC were more likely to be older, had Medicare/Medicaid insurance plans, had lower median household income, had longer hospital stays during their index hospitalization, had higher Frailty Risk Score, and had higher burden of medical comorbidities (Table 1). In addition, HNHC patients were significantly more likely to have an unplanned index hospitalization, more likely to need inpatient glucocorticoids, more likely to undergo IBD-related procedures, and had a severe IBD-related hospitalization (defined by a LOS greater than 7 days or need for IBD-related surgery) (Table 1).

**Table 1.** Baseline characteristics between non-high-need high-cost and high-need high-cost patients at index hospitalization

Characteristics	Non-high-need high-cost patients (n = 42,685)	High-need high-cost patients (n = 4,717)	*P* value
Patient characteristics			
Age (mean ± SD)	53.2 ± 19.4	54.9 ± 18.7	<0.01
Age by categories (%)			<0.01
Age <40	29%	24%	
Age 40–64	39%	41%	
Age >64	32%	35%	
Female (%)	57%	56%	0.06
Primary expected payer			<0.01
Medicare/Medicaid	50%	66%	
Private insurance	40%	26%	
Self-pay	5%	4%	
No charge/others	5%	4%	
Median household income			<0.01
0–25th percentile (≥ $37,999)	22%	26%	
26th–50th percentile ($38,000–$47,999)	25%	26%	
51st–75th percentile ($48,000–$63,999)	26%	24%	
76th–100th percentile (≤$64,000)	26%	24%	
Initial length of stay (in d) (median [range])	3 (2–6)	9 (4–19)	<0.01
Frailty risk categories			<0.01
Low	70%	46%	
Medium/high	30%	54%	
Congestive heart failure (%)	6%	13%	<0.01
Chronic lung disease (%)	18%	22%	<0.01
Diabetes (%)	17%	24%	<0.01
Obesity (%)	9%	11%	<0.01
Peripheral vascular disease (%)	4%	6%	<0.01
Depression (%)	14%	16%	<0.01
Chronic pain (%)	2%	4%	<0.01
Renal failure (%)	9%	17%	<0.01
Charlson Comorbidity Index (%)			<0.01
0	62%	45%	
1	18%	19%	
2+	20%	36%	
Hospital factors			
Urban (%)	8%	5%	<0.01
Teaching status (%)			<0.01
Metropolitan nonteaching	41%	38%	
Metropolitan teaching	51%	57%	
Nonmetropolitan	8%	5%	
Bed size (%)			<0.01
Small	11%	7%	
Medium	24%	22%	
Large	66%	71%	
Hospitalization factors			
Unplanned admission (%)	81%	88%	<0.01
Glucocorticoid use (%)	5%	6%	0.01
IBD-related procedures	28%	41%	<0.01
Severe IBD hospitalization (length of stay > 7 d or need for IBD-related surgery) (%)	14%	55%	<0.01

### Simplified tree-based algorithms and LR model

#### Primary outcome—risk of progressing to HNHC

**Figure 2.**
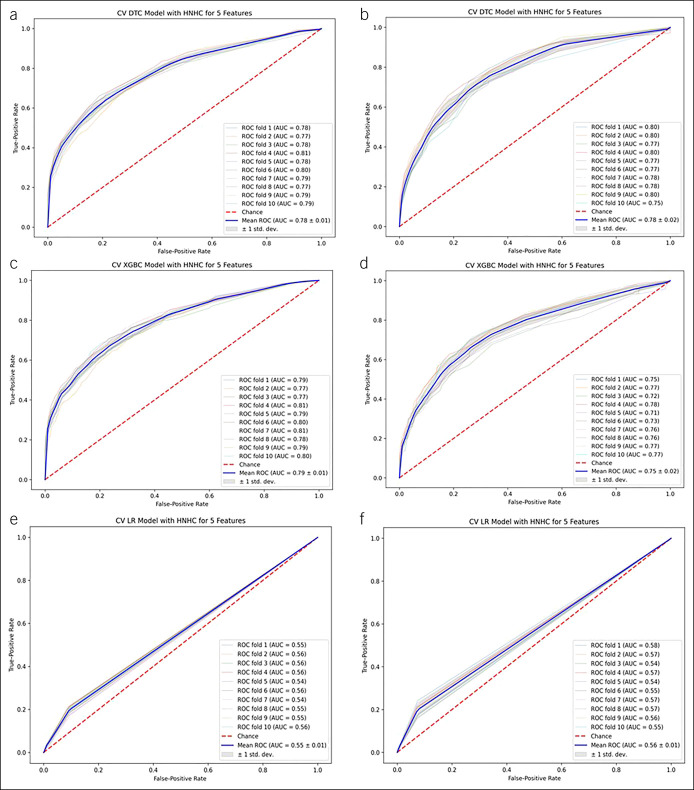
Tree-based and logistic regression models (5 features) for predicting risk of progression to high-need, high-cost patients.

The predictive performance of our simplified models is summarized in Table 2. Both tree-based algorithms outperformed traditional LR in our derivation and validation cohorts. For our derivation cohort, the mean AUC (SD) for DTC was 0.78 (0.01), for XGBoost was 0.79 (0.01), and for LR was 0.55 (0.01) (Figure 2a,c, and e, respectively). The best features for DTC were LOS, Charlson Comorbidity Index, procedure occurring in the operating room, Frailty Risk Score, and age at admission. For XGBoost, the best features were LOS, procedure occurring in the operating room, medical comorbidity with chronic pain, complications from diabetes, and medical comorbidity with drug abuse. For LR, the best features that were selected were medical comorbidity with peptic ulcer without bleeding, medical comorbidity with paresthesia, admission for osteomyelitis, medical comorbidity with renal failure, and medical comorbidity with lymphoma. The variable importance plots for all 3 models are presented in Supplementary Digital Content (see Figure 2A, C, and E, http://links.lww.com/CTG/A827). Across 10-fold cross-validation, the precision of DTC, XGBoost, and LR ranged from 0.76 to 0.85, 0.75 to 0.85, and 0.0, respectively; the accuracy of DTC, XGBoost, and LR ranged from 0.912 to 0.920, 0.910 to 0.920, and 0.90, respectively (Table 2).

**Table 2.** Performance of tree-based machine learning models compared with a traditional logistic regression model to predict progression to HNHC using 5 features at index hospitalization

No. of folds	Decision tree classifier (DTC)				Machine learning model (XGBoost)				Logistic regression			
	Area under curve	Precision	Recall	Accuracy	Area under curve	Precision	Recall	Accuracy	Area under curve	Precision	Recall	Accuracy
Derivation												
1	0.784	0.826	0.201	0.916	0.787	0.808	0.206	0.917	0.547	0.0	0.0	0.900
2	0.770	0.767	0.167	0.912	0.770	0.754	0.182	0.913	0.560	0.0	0.0	0.900
3	0.777	0.821	0.214	0.917	0.774	0.816	0.236	0.919	0.559	0.0	0.0	0.900
4	0.806	0.850	0.240	0.920	0.808	0.830	0.248	0.920	0.561	0.0	0.0	0.900
5	0.780	0.810	0.189	0.915	0.787	0.800	0.195	0.915	0.540	0.0	0.0	0.900
6	0.795	0.858	0.218	0.919	0.799	0.821	0.233	0.919	0.562	0.0	0.0	0.900
7	0.787	0.760	0.235	0.916	0.805	0.848	0.225	0.910	0.540	0.0	0.0	0.900
8	0.772	0.800	0.263	0.920	0.784	0.850	0.239	0.920	0.555	0.0	0.0	0.900
9	0.787	0.767	0.258	0.918	0.793	0.816	0.254	0.920	0.546	0.0	0.0	0.900
10	0.789	0.776	0.235	0.917	0.802	0.820	0.242	0.919	0.555	0.0	0.0	0.900
Validation												
1	0.798	0.800	0.057	0.967	0.751	0.750	0.064	0.967	0.584	1	0.007	0.965
2	0.799	0.864	0.136	0.969	0.773	0.810	0.121	0.0968	0.568	0	0	0.965
3	0.767	1	0.050	0.967	0.716	0.727	0.057	0.966	0.543	0	0	0.965
4	0.802	0.818	0.064	0.967	0.777	0.923	0.086	0.968	0.570	0	0	0.965
5	0.771	0.667	0.057	0.966	0.714	0.917	0.079	0.968	0.545	0	0	0.965
6	0.766	0.923	0.085	0.968	0.732	1	0.085	0.968	0.552	0	0	0.965
7	0.779	0.857	0.085	0.967	0.758	0.769	0.071	0.967	0.570	0	0	0.965
8	0.780	0.667	0.043	0.966	0.762	0.667	0.057	0.966	0.574	0	0	0.965
9	0.805	1	0.086	0.968	0.773	0.846	0.079	0.967	0.565	0	0	0.965
10	0.745	0.818	0.064	0.967	0.770	0.786	0.079	0.967	0.550	1	0.007	0.965

In the external validation cohort, the performance of our models were similar to the performances in the derivation cohort with mean AUC for DTC 0.78 (0.02), XGBoost 0.75 (0.02), and LR 0.56 (0.01) (Figure 2b,d and f, respectively). Table 2 lists the performance metrics of the models in the validation cohort. The variable importance plots for all 3 models are presented in Supplementary Digital Content (see Figure 2B, D, and F, http://links.lww.com/CTG/A827).

On analysis with exclusion of LOS (since that was the variable of highest importance), tree-based algorithms were superior to LR (see Supplementary Figure 3A–F, http://links.lww.com/CTG/A827). Supplementary Digital Content (see Table 1, http://links.lww.com/CTG/A827) summarizes the performance metrics of all 3 models in the derivation and validation cohorts while Supplementary Digital Content (see Figure 4, http://links.lww.com/CTG/A827) presents the variable importance plots. With expansion to 10 features, there was no incremental improvement in predictive accuracy over 5 features; tree-based algorithms continued to outperform LR (see Supplementary Figure 5A–F, Supplementary Table 2, http://links.lww.com/CTG/A827). Corresponding variable importance plots are shown in Supplementary Digital Content (see Figure 6A–F, http://links.lww.com/CTG/A827).

#### Secondary outcome—risk of 90-day readmission

**Figure 3.**
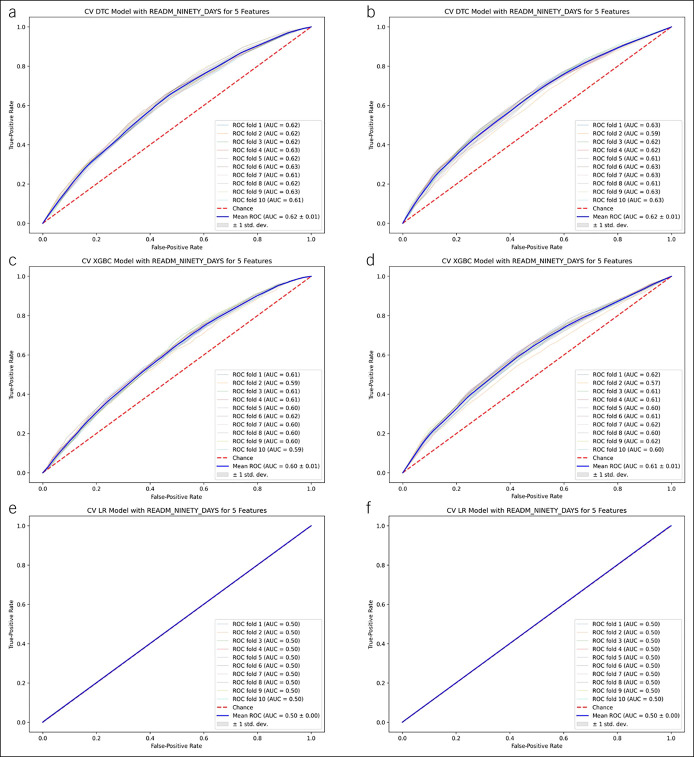
Tree-based and logistic regression models (5 features) for predicting risk of 90-day readmission.

The predictive performance of our simplified ML models is summarized in Supplementary Digital Content (see Table 4, http://links.lww.com/CTG/A827). Both tree-based algorithms outperformed traditional LR in our derivation and validation cohorts. For our derivation cohort, the mean AUC (SD) for DTC was 0.62 (0.01), for XGBoost was 0.60 (0.01), and for LR was 0.50 (0.0) (Figure 3a,c, and E, respectively). Description of the features that were included in each model and performance metrics for each model are summarized in Supplementary Digital Content (see Table 3, http://links.lww.com/CTG/A827), and the relative importance of each variable for each model can be observed in the variable importance plots (see Supplementary Figure 7A, C, and E, http://links.lww.com/CTG/A827).

In the external validation cohort, the mean AUC (SD) for DTC was 0.62 (0.01), for XGBoost was 0.61 (0.01), and for LR was 0.50 (0) (Figure 3b,d and f, respectively). Description of the features that were included in each model and performance metrics for each model are summarized in Supplementary Digital Content (see Table 4, http://links.lww.com/CTG/A827), and the relative importance of each variable for each model can be observed in the variable importance plots (see Supplementary Figure 7B, D, and F, http://links.lww.com/CTG/A827).

On analysis with inclusion of 10 features, tree-based algorithms performed better than LR with mean AUC (SD) for DTC 0.62 (0.02), XGBoost 0.63 (0.01), and LR 0.55 (0.01) for the derivation cohort. For the validation cohort, the best model was XGBoost with mean AUC 0.93 (0.01), followed by DTC 0.62 (0.01) and LR 0.55 (0.01) (see Supplementary Figure 8A–F, http://links.lww.com/CTG/A827). Description of the features in each model and performance metrics are summarized in Supplementary Digital Content (see Table 4, http://links.lww.com/CTG/A827). The relative importance of each variable for each model can be observed in Supplementary Digital Content (see Figure 9, http://links.lww.com/CTG/A827).

## DISCUSSION

In a nationally representative cohort of hospitalized patients with IBD, simplified tree-based ML models outperformed traditional LR in identifying patients at risk for becoming HNHC over the course of 1 year and 90-day readmission risk. Our ML algorithms were built on prior work on readmission risk and included previously known risk factors associated with unplanned healthcare utilization and becoming HNHC: LOS at index hospitalization, IBD-related procedure occurring during hospitalization, comorbidity burden, and frailty (4,12). Using RFE, we were able to find a limited number of features that could accurately identify patients at risk for becoming HNHC and would allow for future integration into a simple clinical decision support tool for a dynamic, point-of-care risk assessment. Accurately identifying these HNHC patients would allow for the development of targeted population health management strategies to improve health outcomes and reduce healthcare costs (9). Regueiro et al (18) previously demonstrated the effectiveness of a population-based, patient-centered IBD medical home, where patients receive multidisciplinary care championed by a gastroenterologist, in reducing unplanned healthcare utilization and healthcare costs in a subset of patients considered to be high utilizer of healthcare resources.

Previous studies on hospitalization and readmission risk using regression-based models have modest discriminative performance in predicting readmission risk and becoming HNHC, and this may be due to failure to account for the multidimensional aspects of healthcare utilization, inability to leverage the vast amounts of diverse healthcare data, and inability to identify potential nonlinear relationships (4). In our previous work using NRD 2013, we developed a multivariate LR model with backward selection to identify 12 key features (younger age, female sex, IBD-related surgery at index hospitalization, insurance payer, rural location, low median household income, large hospital bed size, higher Charlson Comorbidity Index, lack of smoking, depression, obesity, and certain IBD-related admissions) associated with HNHC patients, and this model had a modest AUC of 0.66 (8). Using our current ML algorithms in the same cohort of patients, we were able to develop improved and simplified models with a smaller number of features, allowing for ease of clinical interpretation and reproducibility in other cohorts, with a good discriminatory function (mean AUC of ∼0.80). In a retrospective cohort study of patients with IBD from the national Veterans Health Administration electronic database, Waljee et al also demonstrated the effectiveness of using a ML approach, random forest, in predicting outcomes compared with a traditional LR model. Using this ML approach, the authors developed a model, with an AUC of 0.85 (95% CI, 0.84–0.85), which performed better than a LR model, with an AUC of 0.68 (95% CI, 0.67–0.68), in predicting a composite outcome of outpatient corticosteroid use and hospitalizations (11). Our study adds to the current literature and demonstrates the superiority of ML-based techniques in predicting risk of progressing to HNHC. In addition, our simplified ML algorithms are easy to understand, adopt, and implement on existing electronic health records to allow for point-of-care risk prediction to improve population health management strategies.

We envision that our simplified models would be deployed as point-of-care assessment in the hospital setting after a patient's initial hospitalization. If patients are identified as at-risk for becoming HNHC, then a transitional care team inclusive of a physician, nurse, and pharmacist can help patients identify potential barriers to healthcare access (such as postdischarge follow-up, medication refills, and rehabilitation programs) and modify risks for recurrent and/or prolonged hospitalization.

The strengths of this study are multiple and attempted to address the previously identified limitations of ML models: (i) We accounted for potential selection bias and undersampling by using a large, national database inclusive of hospitals from urban and rural areas, as well as nonteaching and teaching institutions; (ii) we included a large number of patients and more than 100 variables of interest; (iii) we used tree-based algorithms to develop intuitive and parsimonious models for prediction and easy adoption into existing electronic health records; (iv) we developed our models in accordance to PROBAST recommendations for developing and validating prediction models; and (v) we used an external validation cohort to assess the performance of our models (15,19).

There are some inherent limitations. First, this study was retrospective in nature, which limits our ability to validate the data and account for missing data. Second, we were not able to capture data on other established and key risk factors for hospital readmission: (i) disease activity (no data on endoscopy, laboratory, or imaging), (ii) IBD-related medications (e.g., 5-aminosalicylates, outpatient glucocorticoids, immunosuppressants, and biologics), and (iii) social determinants of health. In addition, we used administrative codes and CCS, which may result in misclassification of IBD diagnosis and other patient-related and hospital-related variables. Furthermore, our study focused on hospitalized patients in the United States that did not include other potential factors that may inform readmission risk, such as outpatient care coordination involving postdischarge follow-up, outpatient clinic visits, medication use, patients who have not been hospitalized, the impact of mental health conditions such as mood and anxiety, and patients outside of the United States where healthcare systems and health standards set by governmental agencies are not similar to those in the United States. Studies have demonstrated the bidirectional gut-brain axis in IBD, and future models including mental health conditions may improve the performance of future ML models (20,21). Finally, while we developed our models in 2013, these models may not perform as well with changes in treatment and management over time; however, the variables that were identified during the derivation process may interact with changes in treatment and management and the models may still perform well in subsequent validation studies.

In summary, simplified tree-based algorithms on available administrative claims data can accurately identify patients with IBD at risk for high burden of unplanned healthcare utilization. Future studies incorporating outpatient healthcare utilization, laboratory parameters, medication use, and social determinants of health, in addition to inpatient characteristics, can further refine this risk prediction. Accurate risk prediction is the first step in population health management to promote value-based care in patients with IBD.

## CONFLICTS OF INTEREST

**Guarantor of the article:** Siddharth Singh, MD, MS.

**Specific author contributions:** Study concept and design: N.H.N., P.C., and S.S. Acquisition of data: N.H.N. and S.S. Analysis and interpretation of data: N.H.N., S.P., J.G., A.C., P.C., and S.S. Drafting of the manuscript: N.H.N. and S.S. Critical revision of the manuscript for important intellectual content: N.H.N., S.P., J.G., A.C., W.J.S., L.O.M., P.C., and S.S. Approval of the final manuscript: N.H.N., S.P., J.G., A.C., W.J.S., L.O.M., P.C., and S.S.

**Financial support:** N.H.N. was supported by NIH/NIDDK (T32DK007202) and NLM (T15LM011271). W.J.S. was partially supported by NIDDK-funded San Diego Digestive Diseases Research Center (P30 DK120515). S.S. was supported by NIH/NIDDK (K23DK117058) and ACG Junior Faculty Development Award. L.O.-M. was funded by NIH grants R01HG011066, R01HL136835, OT2OD026552, and U24LM013755.

**Potential competing interests:** N.H.N.: None to declare. S.P.: None to declare. J.G.: None to declare. ASQ: None to declare. A.C.: None to declare. V.J.: AbbVie, Alimentiv (formerly Robarts Clinical Trials), Arena pharmaceuticals, Asieris, Bristol Myers Squibb, Celltrion, Eli Lilly, Ferring, Fresenius Kabi, Galapagos, GlaxoSmithKline, Genetech, Gilead, Janssen, Merck, Mylan, Pandion, Pendopharm, Pfizer, Reistone Biopharma, Roche, Sandoz, Takeda, and Topivert; speaker's fees from, AbbVie, Ferring, Janssen Pfizer Shire, and Takeda. W.J.S.: research grants from Atlantic Healthcare Limited, Amgen, Genentech, Gilead Sciences, AbbVie, Janssen, Takeda, Lilly, Celgene/Receptos, Pfizer, and Prometheus Laboratories (now Prometheus Biosciences); consulting fees from AbbVie, Allergan, Amgen, Arena Pharmaceuticals, Avexegen Therapeutics, BeiGene, Boehringer Ingelheim, Celgene, Celltrion, Conatus, Cosmo, Escalier Biosciences, Ferring, Forbion, Genentech, Gilead Sciences, Gossamer Bio, Incyte, Janssen, Kyowa Kirin Pharmaceutical Research, Landos Biopharma, Lilly, Oppilan Pharma, Otsuka, Pfizer, Progenity, Prometheus Biosciences (merger of Precision IBD and Prometheus Laboratories), Reistone, Ritter Pharmaceuticals, Robarts Clinical Trials (owned by Health Academic Research Trust, HART), Series Therapeutics, Shire, Sienna Biopharmaceuticals, Sigmoid Biotechnologies, Sterna Biologicals, Sublimity Therapeutics, Takeda, Theravance Biopharma, Tigenix, Tillotts Pharma, UCB Pharma, Ventyx Biosciences, Vimalan Biosciences, and Vivelix Pharmaceuticals; and stock or stock options from BeiGene, Escalier Biosciences, Gossamer Bio, Oppilan Pharma, Prometheus Biosciences (merger of Precision IBD and Prometheus Laboratories), Progenity, Ritter Pharmaceuticals, Ventyx Biosciences, Vimalan Biosciences. Spouse: Opthotech–consultant, stock options; Progenity–consultant, stock; Oppilan Pharma–employee, stock options; Escalier Biosciences–employee, stock options; Prometheus Biosciences (merger of Precision IBD and Prometheus Laboratories)–employee, stock options; Ventyx Biosciences: employee, stock options; Vimalan Biosciences: employee, stock options. L.O.M.: None to declare. S.S.: research grants from AbbVie, Janssen. P.C.: Founder of HyperPlanar.

**Study Highlights**

**WHAT IS KNOWN**

✓ A subset of high-need, high-cost patients with IBD use a disproportionate amount of healthcare resources.
✓ Current prediction models have modest performance in identifying these patients.

**WHAT IS NEW HERE**

✓ Our simplified tree-based algorithms can accurately identify patients hospitalized with IBD for becoming high-need and high-cost.

## Supplementary Material
