# Supplementary material for: Simplified Machine Learning Models Can Accurately Identify High-Need High-Cost Patients With Inflammatory Bowel Disease
Source: Clin Transl Gastroenterol. 2022 Jun 7;13(7):e00507. doi: 10.14309/ctg.0000000000000507 (PMC10476830; doi:10.14309/ctg.0000000000000507)
Supplement: Supplementary file 1 [file ct9-13-e00507-s001.docx]

**eTable 1.** Performance of tree-based machine learning models compared to traditional logistic regression model to predict progression to HNHC using five features at index hospitalization after excluding length of hospital stay variable.

| **Number of folds** | **Decision Tree Classifier (DTC)** | | | | **Machine Learning Model (XGBoost)** | | | | **Logistic Regression** | | | |
| --- | --- | --- | --- | --- | --- | --- | --- | --- | --- | --- | --- | --- |
|  | **AUC** | **Precision** | **Recall** | **Accuracy** | **AUC** | **Precision** | **Recall** | **Accuracy** | **AUC** | **Precision** | **Recall** | **Accuracy** |
| **Derivation** | | | | | | | | | | | | |
| **1** | 0.749 | 0.479 | 0.072 | 0.899 | 0.753 | 0.737 | 0.030 | 0.902 | 0.703 | 0 | 0 | 0.900 |
| **2** | 0.724 | 0.390 | 0.034 | 0.899 | 0.731 | 0.362 | 0.036 | 0.898 | 0.674 | 0.308 | 0.008 | 0.899 |
| **3** | 0.741 | 0.513 | 0.083 | 0.901 | 0.759 | 0.690 | 0.042 | 0.903 | 0.696 | 0 | 0 | 0.901 |
| **4** | 0.767 | 0.656 | 0.045 | 0.903 | 0.763 | 0.370 | 0.021 | 0.899 | 0.731 | 0 | 0 | 0.900 |
| **5** | 0.737 | 0.600 | 0.064 | 0.903 | 0.741 | 0.474 | 0.019 | 0.900 | 0.713 | 0 | 0 | 0.900 |
| **6** | 0.767 | 0.484 | 0.032 | 0.900 | 0.786 | 0.500 | 0.023 | 0.900 | 0.720 | 0 | 0 | 0.900 |
| **7** | 0.774 | 0.565 | 0.028 | 0.901 | 0.772 | 0.682 | 0.032 | 0.902 | 0.720 | 0.200 | 0.002 | 0.899 |
| **8** | 0.752 | 0.722 | 0.028 | 0.902 | 0.750 | 0.485 | 0.034 | 0.900 | 0.696 | 0 | 0 | 0.900 |
| **9** | 0.744 | 0.551 | 0.104 | 0.902 | 0.747 | 0.486 | 0.036 | 0.900 | 0.706 | 0.444 | 0.008 | 0.900 |
| **10** | 0.768 | 0.521 | 0.078 | 0.901 | 0.758 | 0.429 | 0.051 | 0.899 | 0.711 | 0 | 0 | 0.899 |
| **Validation** | | | | | | | | | | | | |
| **1** | 0.760 | 0 | 0 | 0.965 | 0.746 | 0 | 0 | 0.965 | 0.679 | 0 | 0 | 0.965 |
| **2** | 0.748 | 0 | 0 | 0.965 | 0.755 | 0 | 0 | 0.965 | 0.699 | 0 | 0 | 0.965 |
| **3** | 0.732 | 0.25 | 0.007 | 0.965 | 0.718 | 0 | 0 | 0.965 | 0.656 | 0 | 0 | 0.965 |
| **4** | 0.780 | 0 | 0 | 0.965 | 0.743 | 0 | 0 | 0.965 | 0.694 | 0 | 0 | 0.965 |
| **5** | 0.745 | 0 | 0 | 0.965 | 0.687 | 0 | 0 | 0.965 | 0.654 | 0 | 0 | 0.965 |
| **6** | 0.721 | 0 | 0 | 0.965 | 0.720 | 0 | 0 | 0.965 | 0.653 | 0 | 0 | 0.965 |
| **7** | 0.757 | 0 | 0 | 0.965 | 0.732 | 0 | 0 | 0.965 | 0.684 | 0 | 0 | 0.965 |
| **8** | 0.746 | 0 | 0 | 0.965 | 0.735 | 0 | 0 | 0.965 | 0.669 | 0 | 0 | 0.965 |
| **9** | 0.749 | 0 | 0 | 0.965 | 0.732 | 0 | 0 | 0.965 | 0.686 | 0 | 0 | 0.965 |
| **10** | 0.740 | 0 | 0 | 0.965 | 0.738 | 0 | 0 | 0.965 | 0.686 | 0 | 0 | 0.965 |

**eTable 2.** Performance of tree-based machine learning models compared to traditional logistic regression model to predict progression to HNHC using 10 features at index hospitalization.

| **Number of folds** | **Decision Tree Classifier (DTC)** | | | | **Machine Learning Model (XGBoost)** | | | | **Logistic Regression** | | | |
| --- | --- | --- | --- | --- | --- | --- | --- | --- | --- | --- | --- | --- |
|  | **AUC** | **Precision** | **Recall** | **Accuracy** | **AUC** | **Precision** | **Recall** | **Accuracy** | **AUC** | **Precision** | **Recall** | **Accuracy** |
| **Derivation** | | | | | | | | | | | | |
| **1** | 0.785 | 0.862 | 0.172 | 0.919 | 0.789 | 0.762 | 0.210 | 0.915 | 0.601 | 0 | 0 | 0.900 |
| **2** | 0.767 | 0.767 | 0.167 | 0.919 | 0.788 | 0.676 | 0.195 | 0.911 | 0.591 | 0 | 0 | 0.900 |
| **3** | 0.773 | 0.823 | 0.217 | 0.919 | 0.792 | 0.777 | 0.244 | 0.918 | 0.612 | 0 | 0 | 0.901 |
| **4** | 0.803 | 0.788 | 0.285 | 0.918 | 0.804 | 0.750 | 0.236 | 0.916 | 0.610 | 0 | 0 | 0.900 |
| **5** | 0.780 | 0.811 | 0.191 | 0.919 | 0.783 | 0.760 | 0.208 | 0.915 | 0.577 | 0 | 0 | 0.900 |
| **6** | 0.791 | 0.857 | 0.216 | 0.919 | 0.812 | 0.809 | 0.242 | 0.919 | 0.607 | 0.500 | 0.002 | 0.901 |
| **7** | 0.787 | 0.870 | 0.212 | 0.919 | 0.803 | 0.842 | 0.237 | 0.920 | 0.579 | 1.000 | 0.002 | 0.901 |
| **8** | 0.772 | 0.894 | 0.197 | 0.918 | 0.789 | 0.806 | 0.237 | 0.918 | 0.608 | 1.000 | 0.002 | 0.900 |
| **9** | 0.792 | 0.819 | 0.258 | 0.919 | 0.803 | 0.776 | 0.256 | 0.918 | 0.605 | 0 | 0 | 0.900 |
| **10** | 0.791 | 0.739 | 0.246 | 0.919 | 0.806 | 0.774 | 0.255 | 0.918 | 0.609 | 0 | 0 | 0.900 |
| **Validation** | | | | | | | | | | | | |
| **1** | 0.795 | 0.750 | 0.064 | 0.967 | 0.789 | 0.800 | 0.057 | 0.967 | 0.629 | 1 | 0.001 | 0.965 |
| **2** | 0.775 | 0.941 | 0.114 | 0.969 | 0.806 | 0.818 | 0.129 | 0.969 | 0.670 | 0 | 0.002 | 0.965 |
| **3** | 0.766 | 1 | 0.050 | 0.967 | 0.784 | 0.571 | 0.057 | 0.966 | 0.619 | 0 | 0.002 | 0.965 |
| **4** | 0.800 | 0.818 | 0.64 | 0.967 | 0.810 | 0.929 | 0.093 | 0.968 | 0.663 | 1 | 0.001 | 0.965 |
| **5** | 0.769 | 0.667 | 0.57 | 0.966 | 0.778 | 0.706 | 0.086 | 0.967 | 0.613 | 0 | 0.002 | 0.965 |
| **6** | 0.763 | 1 | 0.064 | 0.967 | 0.787 | 0.875 | 0.099 | 0.968 | 0.27 | 0 | 0.002 | 0.965 |
| **7** | 0.777 | 0.857 | 0.085 | 0.967 | 0.798 | 0.765 | 0.092 | 0.967 | 0.676 | 0 | 0.002 | 0.965 |
| **8** | 0.769 | 0.667 | 0.043 | 0.966 | 0.811 | 0.533 | 0.057 | 0.965 | 0.638 | 0 | 0.002 | 0.965 |
| **9** | 0.800 | 1 | 0.086 | 0.968 | 0.810 | 0.750 | 0.086 | 0.967 | 0.655 | 0 | 0.002 | 0.965 |
| **10** | 0.740 | 1 | 0.064 | 0.967 | 0.797 | 0.733 | 0.079 | 0.967 | 0.619 | 0 | 0.002 | 0.965 |

**eTable 3.** Performance of tree-based machine learning models compared to traditional logistic regression model to predict 90-day readmission using 5 features at index hospitalization.

| **Number of folds** | **Decision Tree Classifier (DTC)** | | | | **Machine Learning Model (XGBoost)** | | | | **Logistic Regression** | | | |
| --- | --- | --- | --- | --- | --- | --- | --- | --- | --- | --- | --- | --- |
|  | **AUC** | **Precision** | **Recall** | **Accuracy** | **AUC** | **Precision** | **Recall** | **Accuracy** | **AUC** | **Precision** | **Recall** | **Accuracy** |
| **Derivation** | | | | | | | | | | | | |
| **1** | 0.620 | 0.450 | 0.006 | 0.690 | 0.609 | 0.444 | 0.008 | 0.690 | 0.501 | 0.50 | 0.001 | 0.691 |
| **2** | 0.617 | 0.532 | 0.017 | 0.691 | 0.594 | 0.448 | 0.009 | 0.690 | 0.50 | 0 | 0 | 0.691 |
| **3** | 0.617 | 0.440 | 0.038 | 0.687 | 0.611 | 0.347 | 0.012 | 0.687 | 0.501 | 0.25 | 0.001 | 0.691 |
| **4** | 0.626 | 0.500 | 0.016 | 0.691 | 0.608 | 0.353 | 0.008 | 0.688 | 0.501 | 0.50 | 0.001 | 0.691 |
| **5** | 0.624 | 0.421 | 0.011 | 0.689 | 0.601 | 0.514 | 0.012 | 0.691 | 0.501 | 1.00 | 0.001 | 0.691 |
| **6** | 0.629 | 0.429 | 0.004 | 0.690 | 0.616 | 0.467 | 0.010 | 0.690 | 0.503 | 1.00 | 0.001 | 0.691 |
| **7** | 0.610 | 0.475 | 0.013 | 0.690 | 0.604 | 0.333 | 0.010 | 0.687 | 0.500 | 1.00 | 0.001 | 0.691 |
| **8** | 0.622 | 0.452 | 0.045 | 0.687 | 0.602 | 0.426 | 0.010 | 0.689 | 0.501 | 0 | 0 | 0.691 |
| **9** | 0.635 | 0.489 | 0.016 | 0.690 | 0.604 | 0.459 | 0.012 | 0.690 | 0.502 | 0.75 | 0.002 | 0.691 |
| **10** | 0.608 | 0.478 | 0.008 | 0.690 | 0.594 | 0.217 | 0.003 | 0.688 | 0.501 | 0.50 | 0.001 | 0.691 |
| **Validation** | | | | | | | | | | | | |
| **1** | 0.631 | 0.552 | 0.670 | 0.644 | 0.620 | 0.551 | 0.165 | 0.640 | 0.500 | 0.333 | 0.001 | 0.628 |
| **2** | 0.595 | 0.491 | 0.232 | 0.646 | 0.575 | 0.496 | 0.148 | 0.627 | 0.502 | 0.500 | 0.001 | 0.628 |
| **3** | 0.621 | 0.531 | 0.345 | 0.647 | 0.611 | 0.548 | 0.165 | 0.639 | 0.501 | 0.250 | 0.002 | 0.628 |
| **4** | 0.620 | 0.620 | 0.194 | 0.645 | 0.611 | 0.538 | 0.198 | 0.639 | 0.502 | 1 | 0 | 0.629 |
| **5** | 0.612 | 0.498 | 0.219 | 0.647 | 0.602 | 0.493 | 0.174 | 0.627 | 0.501 | 0.400 | 0.001 | 0.628 |
| **6** | 0.629 | 0.558 | 0.263 | 0.645 | 0.610 | 0.537 | 0.189 | 0.638 | 0.502 | 0 | 0 | 0.628 |
| **7** | 0.630 | 0.569 | 0.263 | 0.644 | 0.618 | 0.552 | 0.175 | 0.641 | 0.501 | 0.500 | 0.001 | 0.628 |
| **8** | 0.614 | 0.541 | 0.271 | 0.646 | 0.599 | 0.532 | 0.160 | 0.636 | 0.502 | 1 | 0 | 0.629 |
| **9** | 0.631 | 0.557 | 0.239 | 0.646 | 0.615 | 0.280 | 0.186 | 0.648 | 0.501 | 0.500 | 0.002 | 0.629 |
| **10** | 0.627 | 0.530 | 0.267 | 0.646 | 0.596 | 0.527 | 0.176 | 0.635 | 0.501 | 0.500 | 0.002 | 0.629 |

**eTable 4.** Performance of tree-based machine learning models compared to traditional logistic regression model to predict 90-day readmission using 10 features at index hospitalization.

| **Number of folds** | **Decision Tree Classifier (DTC)** | | | | **Machine Learning Model (XGBoost)** | | | | **Logistic Regression** | | | |
| --- | --- | --- | --- | --- | --- | --- | --- | --- | --- | --- | --- | --- |
|  | **AUC** | **Precision** | **Recall** | **Accuracy** | **AUC** | **Precision** | **Recall** | **Accuracy** | **AUC** | **Precision** | **Recall** | **Accuracy** |
| **Derivation** | | | | | | | | | | | | |
| **1** | 0.622 | 0.427 | 0.028 | 0.687 | 0.643 | 0.519 | 0.099 | 0.692 | 0.553 | 0.433 | 0.009 | 0.690 |
| **2** | 0.619 | 0.524 | 0.015 | 0.691 | 0.626 | 0.466 | 0.095 | 0.687 | 0.549 | 0.575 | 0.034 | 0.693 |
| **3** | 0.617 | 0.339 | 0.013 | 0.687 | 0.624 | 0.406 | 0.103 | 0.682 | 0.537 | 0.418 | 0.019 | 0.688 |
| **4** | 0.625 | 0.510 | 0.017 | 0.691 | 0.638 | 0.484 | 0.099 | 0.689 | 0.555 | 0.468 | 0.255 | 0.690 |
| **5** | 0.621 | 0.451 | 0.016 | 0.689 | 0.628 | 0.488 | 0.111 | 0.689 | 0.547 | 0.361 | 0.018 | 0.686 |
| **6** | 0.623 | 0.511 | 0.016 | 0.691 | 0.638 | 0.464 | 0.095 | 0.688 | 0.560 | 0.508 | 0.021 | 0.691 |
| **7** | 0.614 | 0.415 | 0.040 | 0.685 | 0.624 | 0.497 | 0.098 | 0.690 | 0.556 | 0.486 | 0.023 | 0.690 |
| **8** | 0.619 | 0.461 | 0.048 | 0.688 | 0.636 | 0.510 | 0.095 | 0.691 | 0.553 | 0.481 | 0.026 | 0.690 |
| **9** | 0.630 | 0.545 | 0.004 | 0.691 | 0.628 | 0.519 | 0.104 | 0.692 | 0.553 | 0.511 | 0.031 | 0.691 |
| **10** | 0.608 | 0.520 | 0.018 | 0.691 | 0.613 | 0.444 | 0.099 | 0.685 | 0.555 | 0.559 | 0.022 | 0.692 |
| **Validation** | | | | | | | | | | | | |
| **1** | 0.627 | 0.552 | 0.214 | 0.643 | 0.942 | 0.836 | 0.903 | 0.898 | 0.553 | 0.563 | 0.046 | 0.633 |
| **2** | 0.598 | 0.507 | 0.200 | 0.630 | 0.931 | 0.827 | 0.891 | 0.890 | 0.550 | 0.569 | 0.035 | 0.630 |
| **3** | 0.621 | 0.535 | 0.235 | 0.640 | 0.929 | 0.817 | 0.879 | 0.882 | 0.550 | 0.581 | 0.030 | 0.627 |
| **4** | 0.621 | 0.563 | 0.188 | 0.644 | 0.945 | 0.833 | 0.908 | 0.898 | 0.545 | 0.565 | 0.039 | 0.631 |
| **5** | 0.608 | 0.491 | 0.210 | 0.626 | 0.930 | 0.822 | 0.885 | 0.886 | 0.549 | 0.568 | 0.040 | 0.630 |
| **6** | 0.625 | 0.549 | 0.236 | 0.644 | 0.932 | 0.826 | 0.881 | 0.887 | 0.558 | 0.561 | 0.050 | 0.636 |
| **7** | 0.632 | 0.568 | 0.259 | 0.652 | 0.934 | 0.819 | 0.886 | 0.885 | 0.557 | 0.575 | 0.034 | 0.630 |
| **8** | 0.611 | 0.518 | 0.259 | 0.635 | 0.929 | 0.821 | 0.880 | 0.884 | 0.559 | 0.570 | 0.035 | 0.632 |
| **9** | 0.629 | 0.550 | 0.246 | 0.645 | 0.937 | 0.821 | 0.889 | 0.887 | 0.555 | 0.565 | 0.041 | 0.632 |
| **10** | 0.624 | 0.531 | 0.235 | 0.639 | 0.936 | 0.822 | 0.899 | 0.890 | 0.564 | 0.559 | 0.038 | 0.634 |

**eFigure 1. Flow diagram for development of derivation cohort.**

**
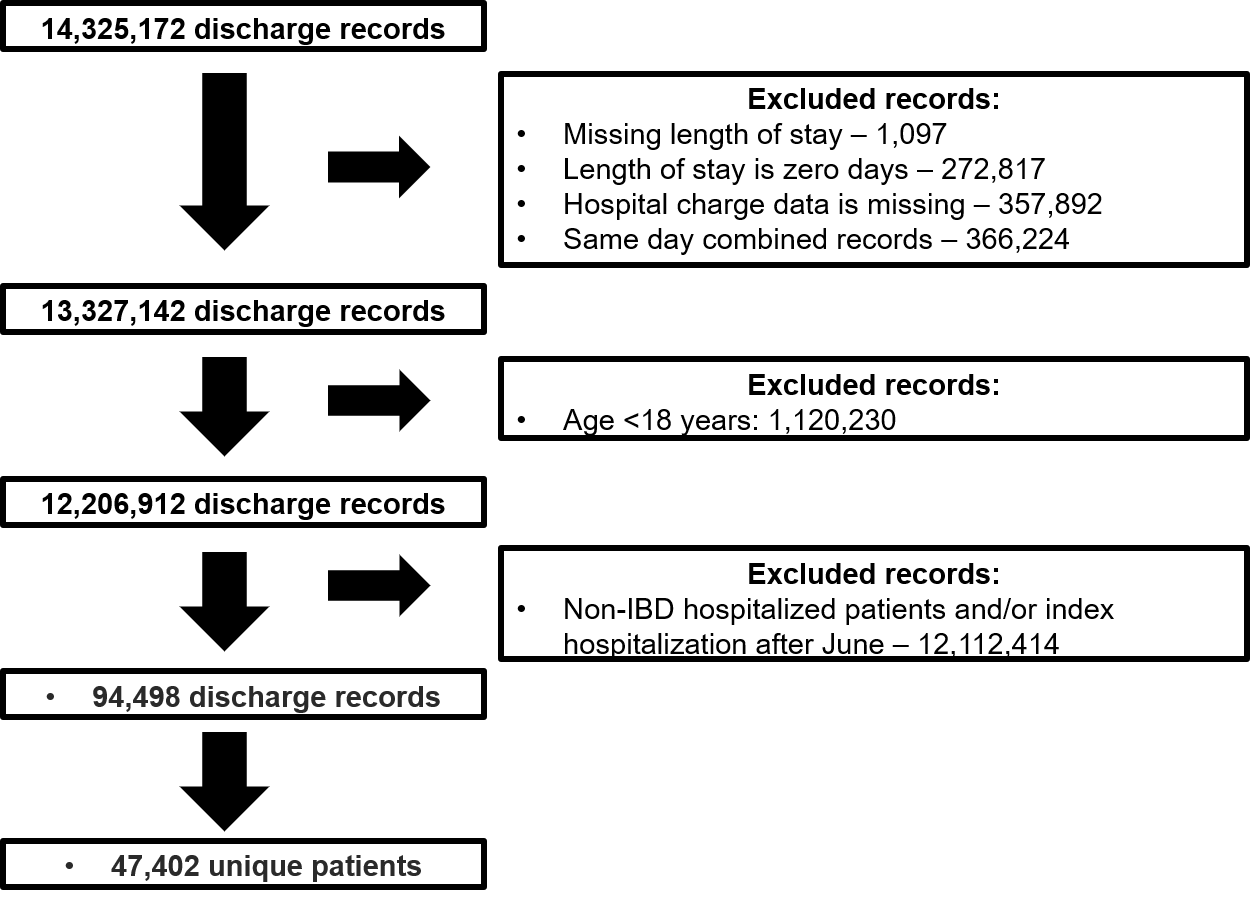
**

**eFigure 2.** Variable importance plots and coefficient weights for tree-based and LR models for predicting risk of progression to high-need, high-cost patients – 5 features.

**
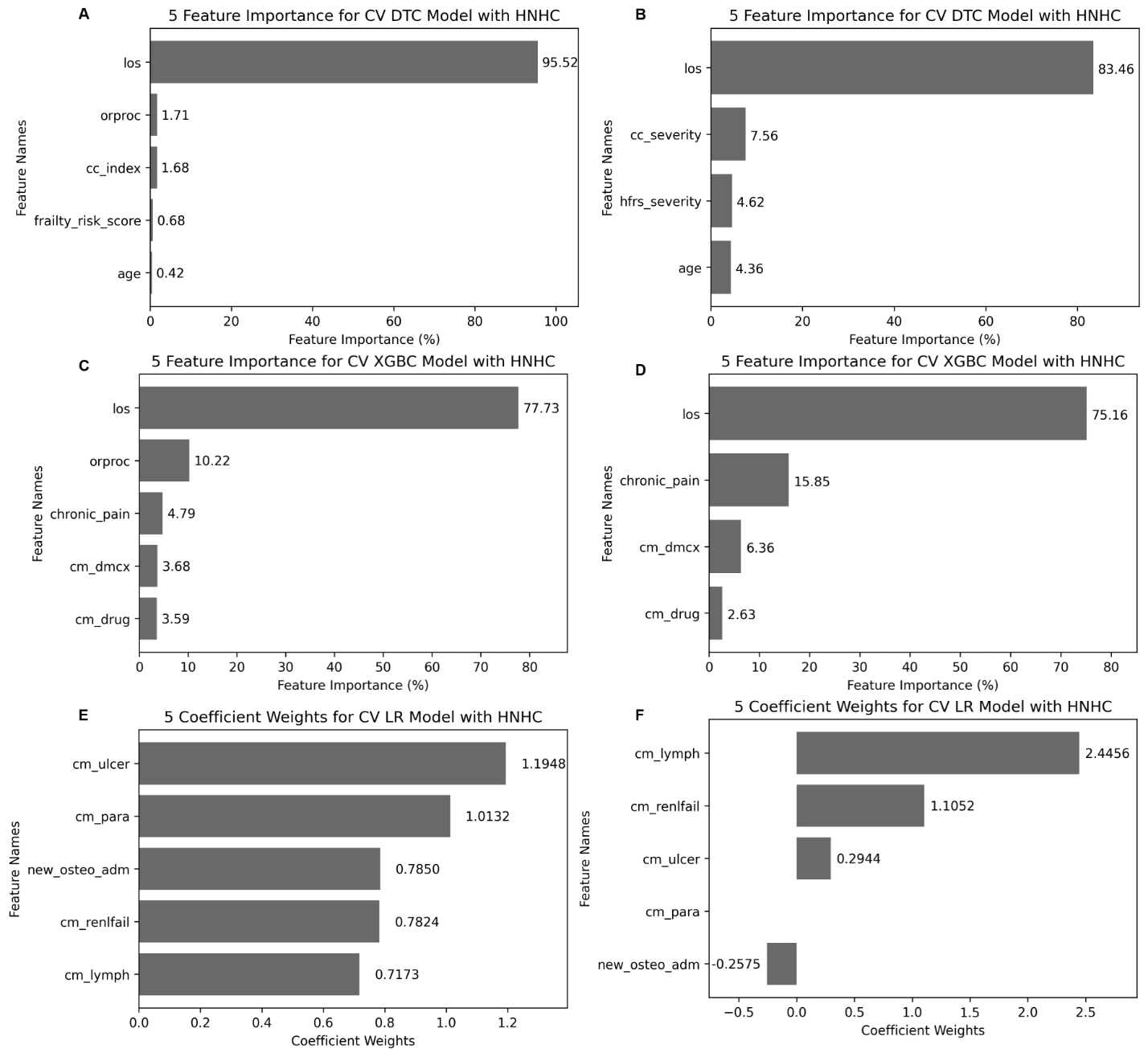
**

**Features included into each model - Variables of importance**

Derivation

- **DTC:** Length of stay, Charlson comorbidity index, procedure occurring in the operating room, frailty risk score, and age
- **XGBoost:** Length of stay, procedure occurring in the operating room, medical comorbidity with chronic pain, complications from diabetes, and medical comorbidity with drug abuse
- **LR:** medical comorbidity with peptic ulcer disease without bleeding, medical comorbidity with paresthesia, admission for osteomyelitis, medical comorbidity with renal failure, and medical comorbidity with lymphoma

Validation

- **DTC**: Length of stay, Charlson comorbidity index, frailty risk score, and age. Of note, we could not cross-walk variable, “procedure occurring in the operating room” since it was not available in NRD 2017
- **XGBoost**: Length of stay, medical comorbidity with chronic pain, complications from diabetes, and medical comorbidity with drug abuse. Of note, we could not cross-walk variable, “procedure occurring in the operating room” since it was not available in NRD 2017
- **LR:** medical comorbidity with peptic ulcer disease without bleeding, medical comorbidity with paresthesia, admission for osteomyelitis, comorbidity with renal failure, and medical comorbidity with lymphoma

**eFigure 3.** Tree-based and logistic regression models, excluding length of stay variable, for predicting risk of progression to high-need, high-cost patients – 5 features.

**
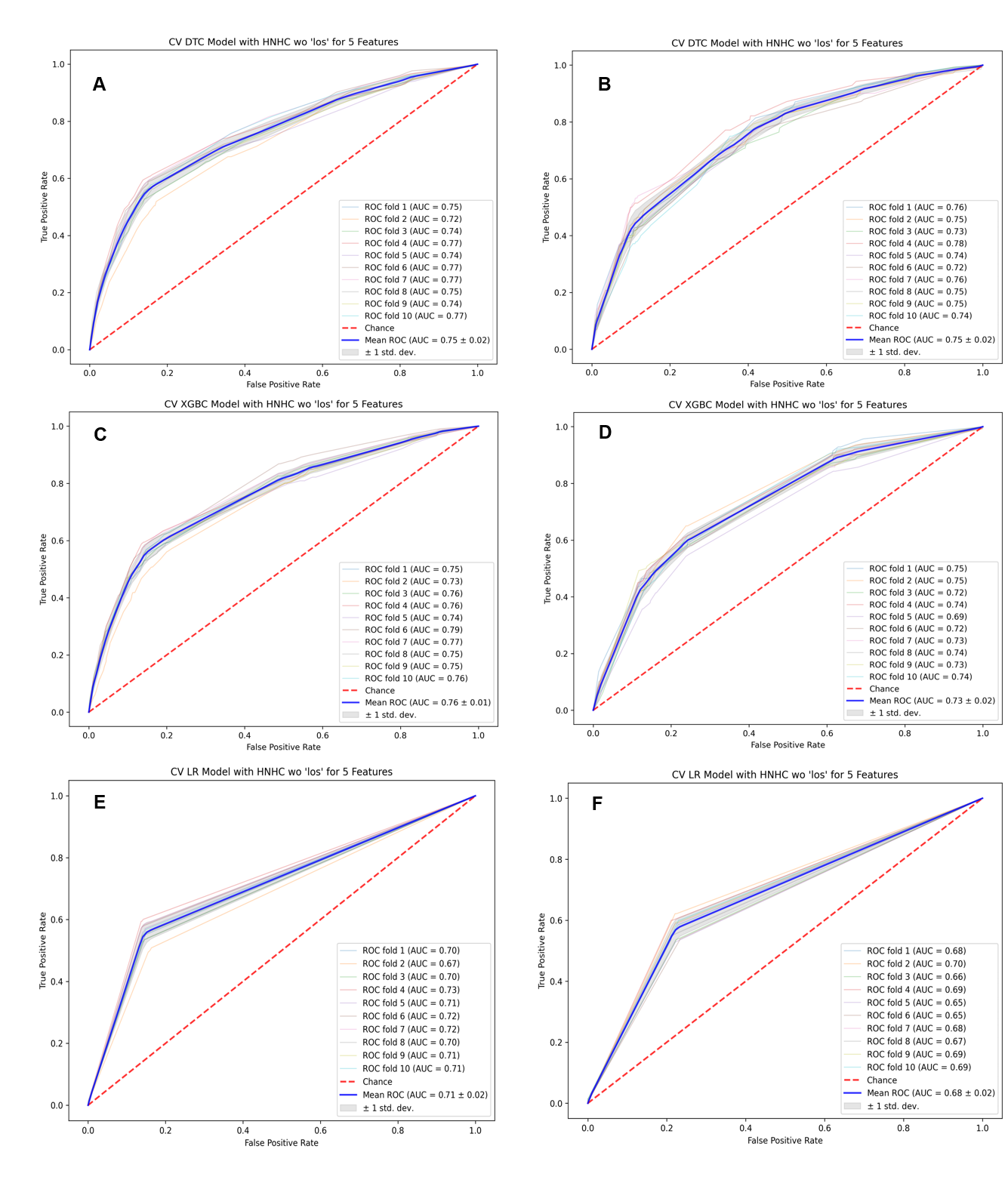
**

**eFigure 4.** Variable importance plots and coefficient weights, with exclusion of length of stay variable, for tree-based and LR models for predicting risk of progression to high-need, high-cost patients – 5 features.


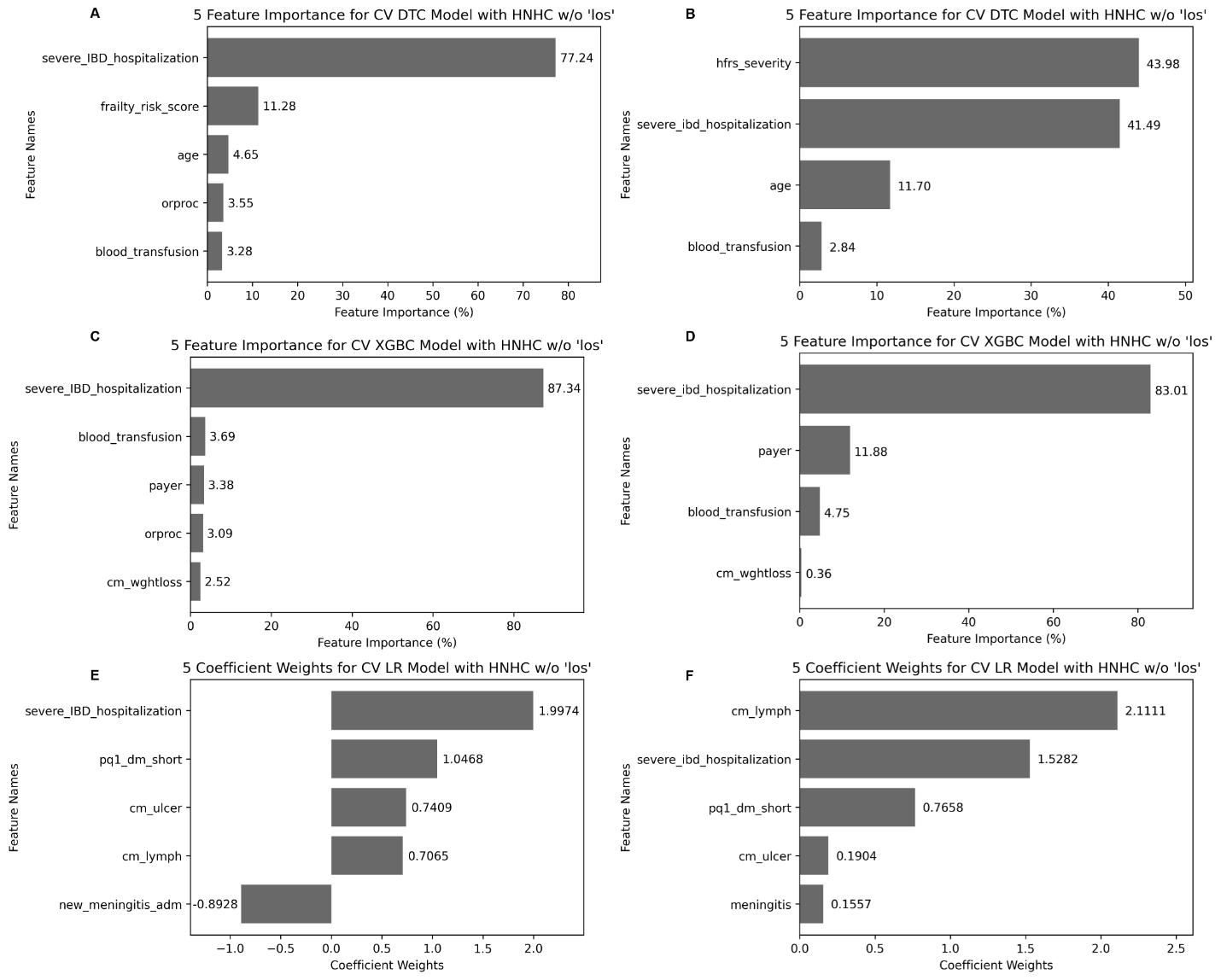


Features included into each model – variables of importance

Derivation

- **DTC:** severe IBD hospitalization (defined as initial length of stay >7 days or IBD-related surgery), frailty risk score, age, procedure occurring in the operating room, and blood transfusion.
- **XGBoost:** severe IBD hospitalization (defined as initial length of stay >7 days or IBD-related surgery), type of insurance payer, blood transfusion, procedure occurring in an operating room, and weight loss as a comorbidity.
- **LR:** severe IBD hospitalization (defined as initial length of stay >7 days or IBD-related surgery), preventable admission for complications related to diabetes, chronic comorbidity with peptic ulcer without bleeding, chronic comorbidity with lymphoma, and admission for meningitis.

Validation

- **DTC:** severe IBD hospitalization (defined as initial length of stay >7 days or IBD-related surgery), frailty risk score, age, and blood transfusion. Of note, we could not cross-walk variable, “procedure occurring in the operating room” since it was not available in NRD 2017
- **XGBoost:** severe IBD hospitalization (defined as initial length of stay >7 days or IBD-related surgery), type of insurance payer, blood transfusion, and weight loss as a comorbidity. Of note, we could not cross-walk variable, “procedure occurring in the operating room” since it was not available in NRD 2017
- **LR:** severe IBD hospitalization (defined as initial length of stay >7 days or IBD-related surgery), preventable admission for complications related to diabetes, chronic comorbidity with peptic ulcer without bleeding, chronic comorbidity with lymphoma, and admission for meningitis.

**eFigure 5.** Tree-based and logistic regression models (10 features) for predicting risk of progression to high-need, high-cost patients.

**
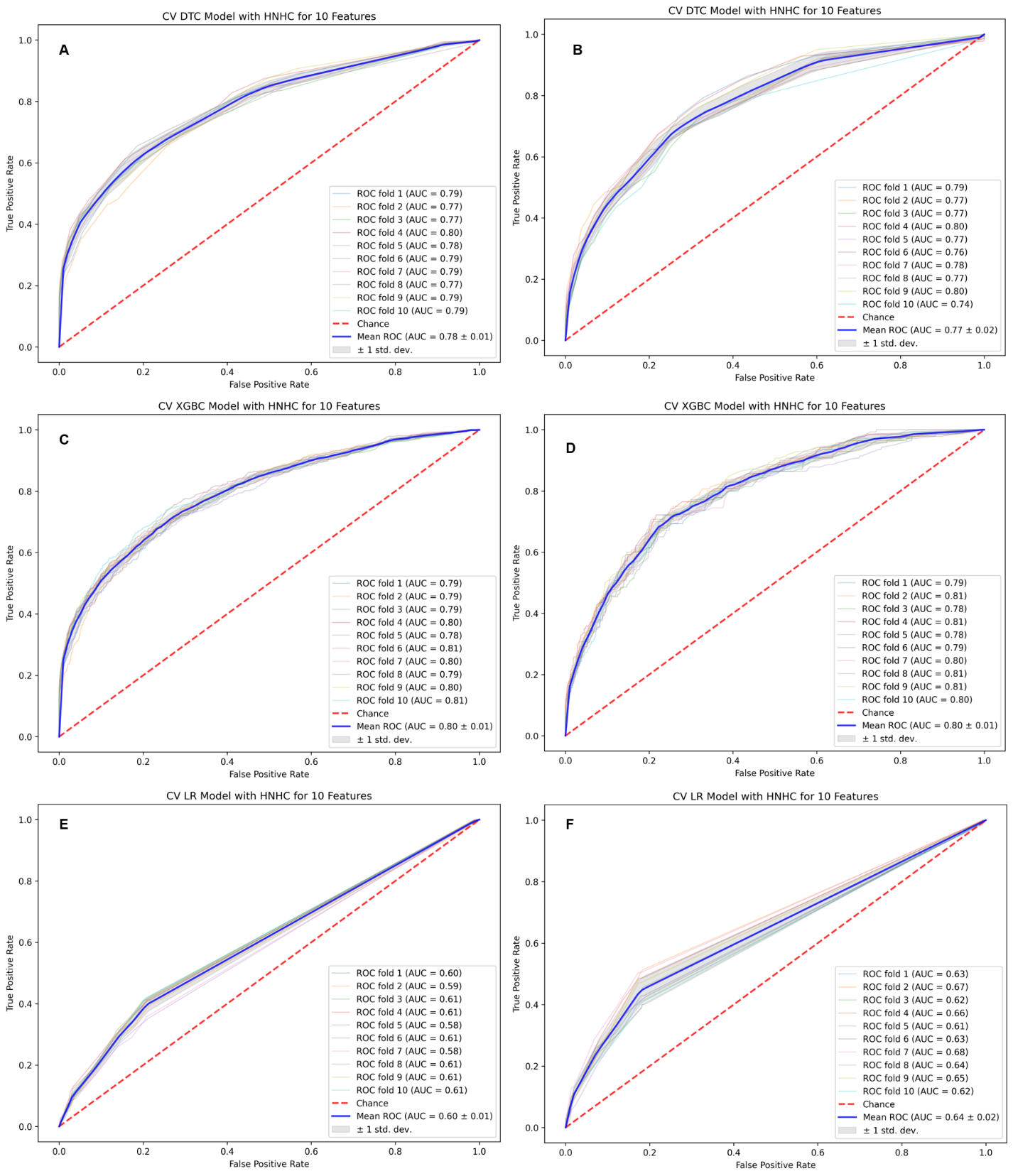
**

**eFigure 6.** Variable importance plots and coefficient weights for tree-based and LR models for predicting risk of progression to high-need, high-cost patients – 10 features.

**
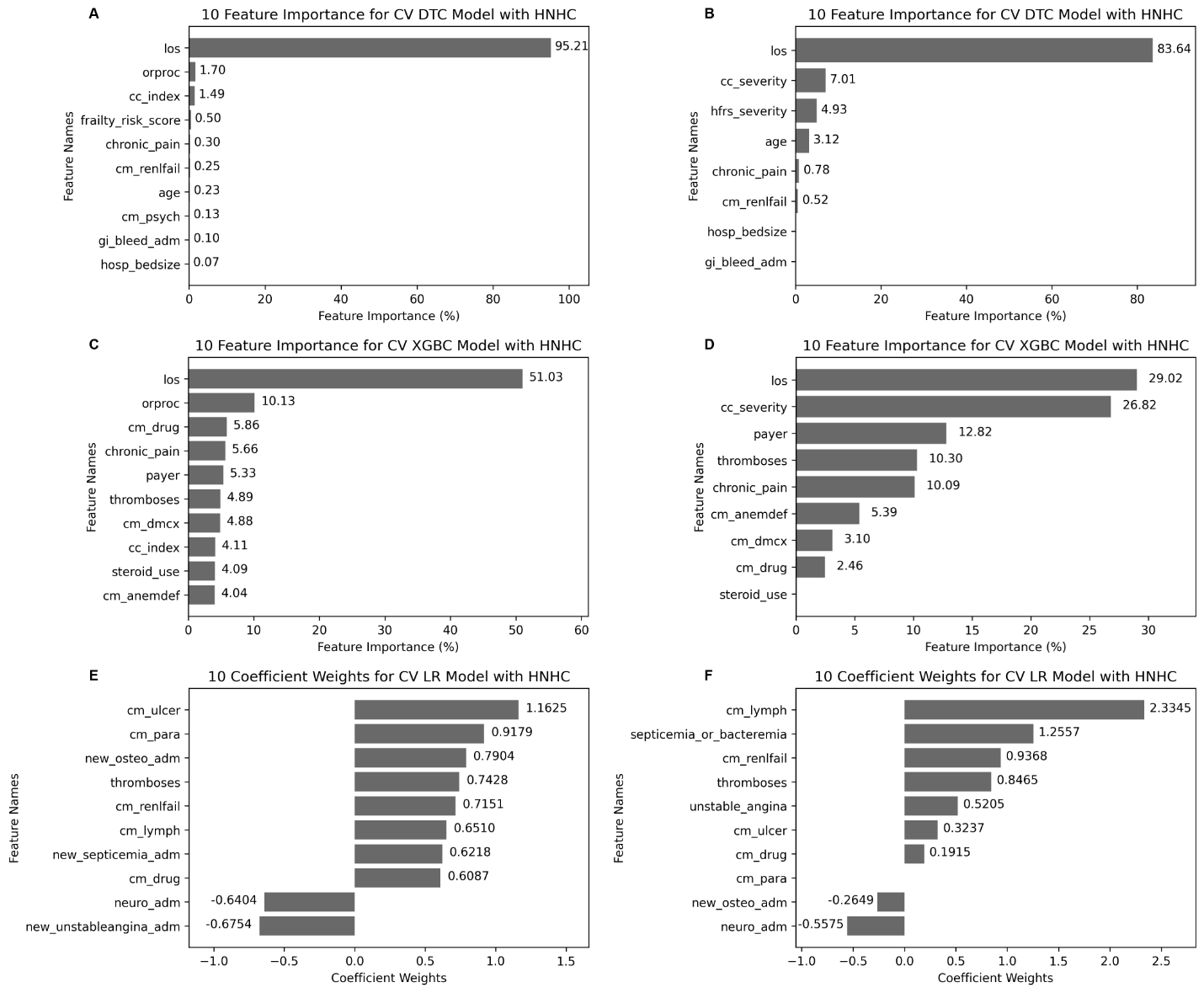
**

Features included into each model - variables of importance

Derivation

- **DTC:** length of stay, procedure occurring in an operating room, Charlson comorbidity index, Frailty risk score, medical comorbidity with chronic pain, medical comorbidity with renal failure, age, medical comorbidity with psychoses, admission for gastrointestinal bleeding, and hospital bed size.
- **XGBoost:** length of stay, procedure occurring in an operating room, medical comorbidity with drug abuse, medical comorbidity with chronic pain, type of insurance payer, admission for thromboembolic events, medical comorbidity with complication related to diseases, Charlson comorbidity index, glucocorticoid use, and medical comorbidity with deficiency anemias.
- **LR:** medical comorbidity with non-bleeding peptic ulcer, medical comorbidity with paresthesia, admission for osteomyelitis, thromboembolic events, medical comorbidity with renal failure, medical comorbidity with lymphoma, admission for septicemia, medical comorbidity with drug abuse, admission for neurologic indication, and admission for unstable angina.

Validation

- **DTC:** length of stay, Charlson comorbidity index, Frailty risk score, medical comorbidity with chronic pain, medical comorbidity with renal failure, age, admission for gastrointestinal bleeding, and hospital bed size. Of note, we could not cross-walk variable, “procedure occurring in the operating room” since it was not available in NRD 2017
- **XGBoost:** length of stay, medical comorbidity with drug abuse, medical comorbidity with chronic pain, type of insurance payer, admission for thromboembolic events, medical comorbidity with complicates related to diseases, Charlson comorbidity index, glucocorticoid use, and medical comorbidity with deficiency anemias. Of note, we could not cross-walk variable, “procedure occurring in the operating room” since it was not available in NRD 2017
- **LR:** medical comorbidity with non-bleeding peptic ulcer, medical comorbidity with paresthesia, admission for osteomyelitis, thromboembolic events, medical comorbidity with renal failure, medical comorbidity with lymphoma, admission for septicemia, medical comorbidity with drug abuse, admission for neurologic indication, and admission for unstable angina.

**eFigure 7.** Variable importance plots and coefficient weights for tree-based and LR models for 90-day readmission risk – 5 features.

**
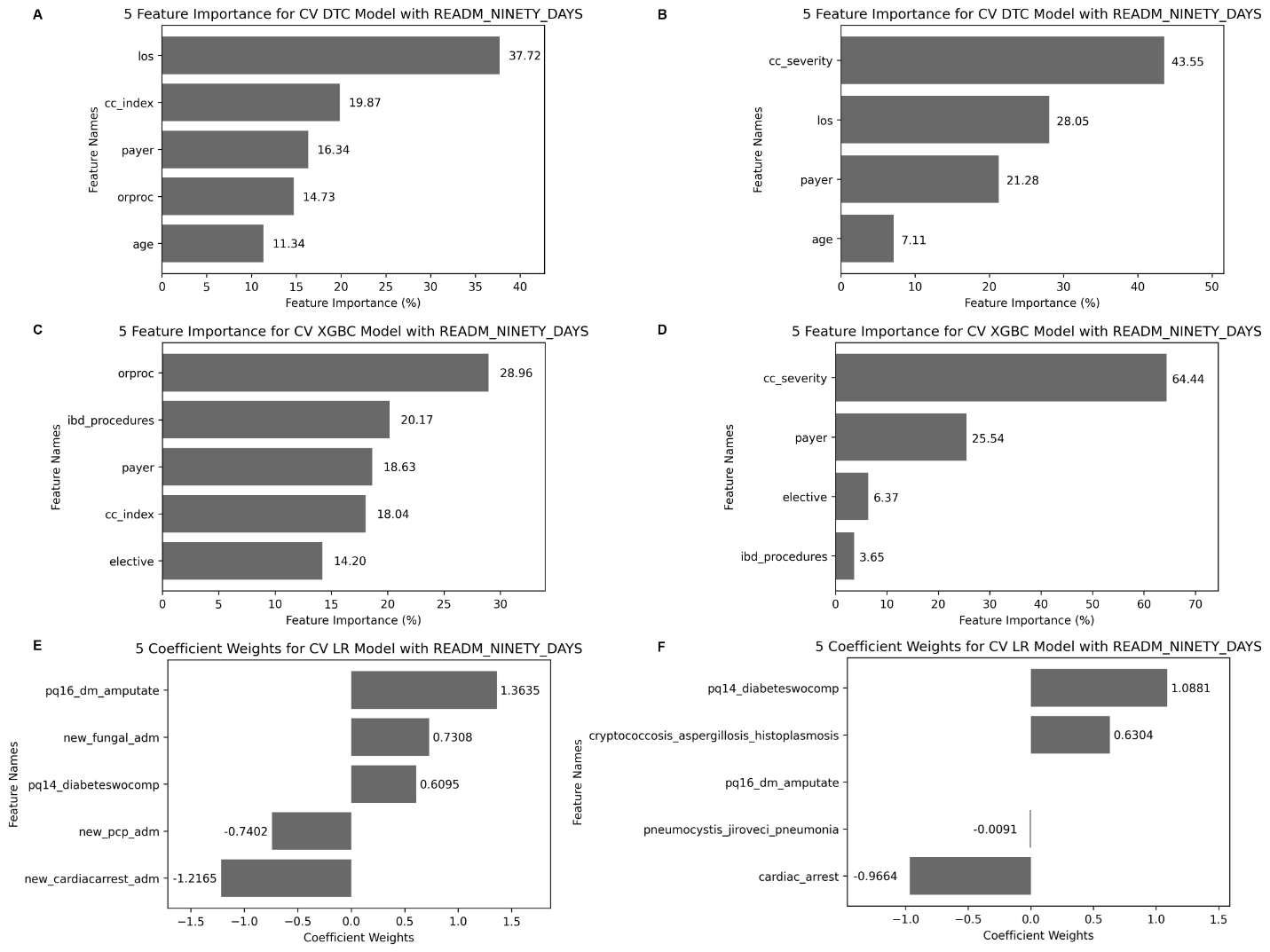
**

Features included into each model – variables of importance

Derivation

- **DTC:** length of stay, Charlson comorbidity index, type of insurance payer, procedure occurring in the operating room, and age.
- **XGBoost:** procedure occurring in the operating room, IBD-related procedures, type of insurance payer, Charlson comorbidity index, and elective vs non-elective admission.
- **LR:** preventable admission for amputation related to diabetes, admission for a fungal infection, preventable admission for diabetes without accompanying complications, admission for pneumocystis jiroveci pneumonia, and new admission for cardiac arrest.

Validation

- **DTC:** length of stay, Charlson comorbidity index, insurance payer, and age.
- **XGBoost:** IBD-related procedures, type of insurance payer, Charlson comorbidity index, and elective vs non-elective admission. Of note, we could not cross-walk variable, “procedure occurring in the operating room” since it was not available in NRD 2017
- **LR:** preventable admission for amputation related to diabetes, admission for a fungal infection, preventable admission for diabetes without accompanying complications, admission for pneumocystis jiroveci pneumonia, and new admission for cardiac arrest.

**eFigure 8.** Tree-based and logistic regression models (10 features) for predicting risk of 90-day readmission.


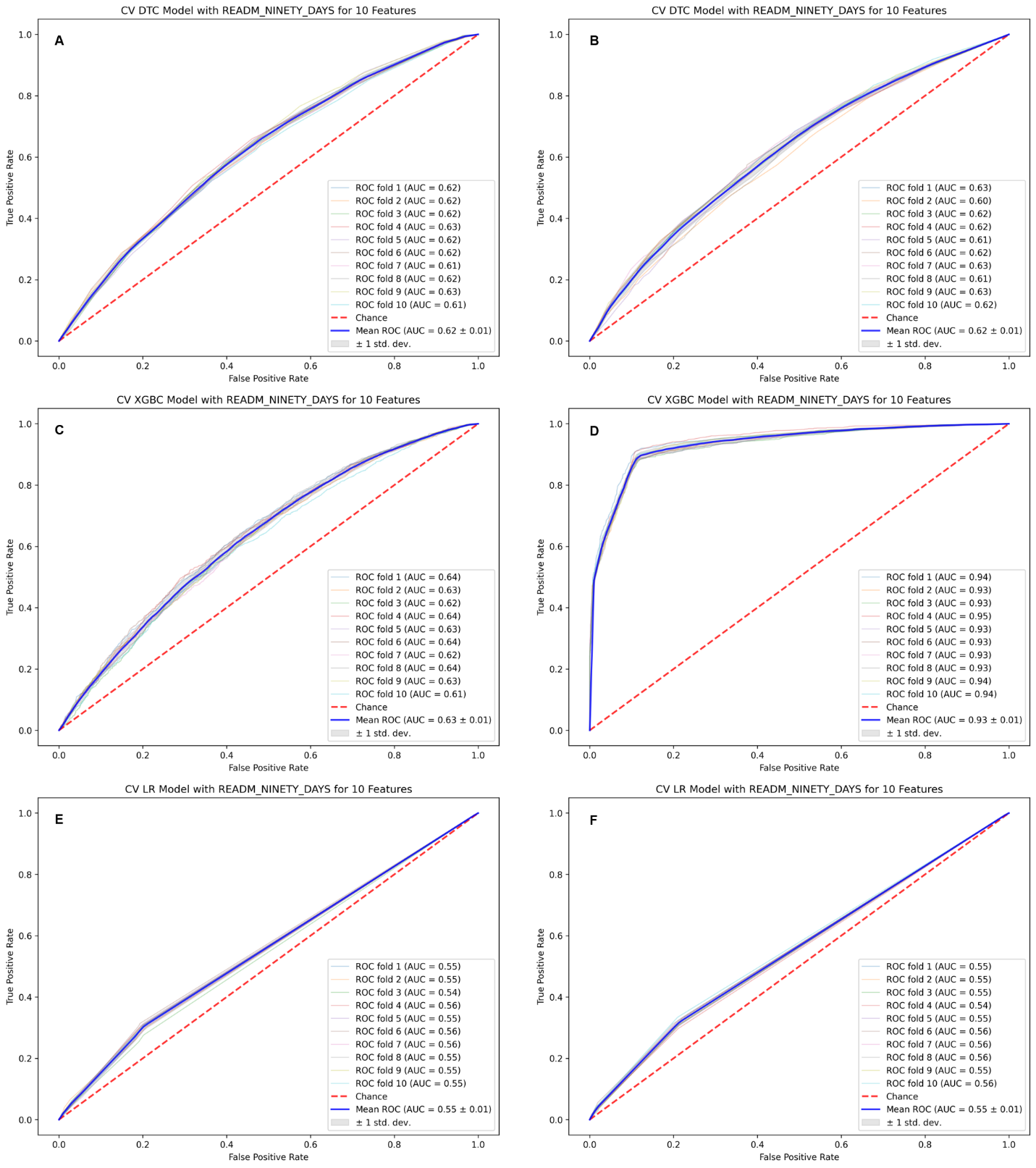


**eFigure 9.** Variable importance plots and coefficient weights for tree-based and LR models for 90-day readmission risk – 10 features.


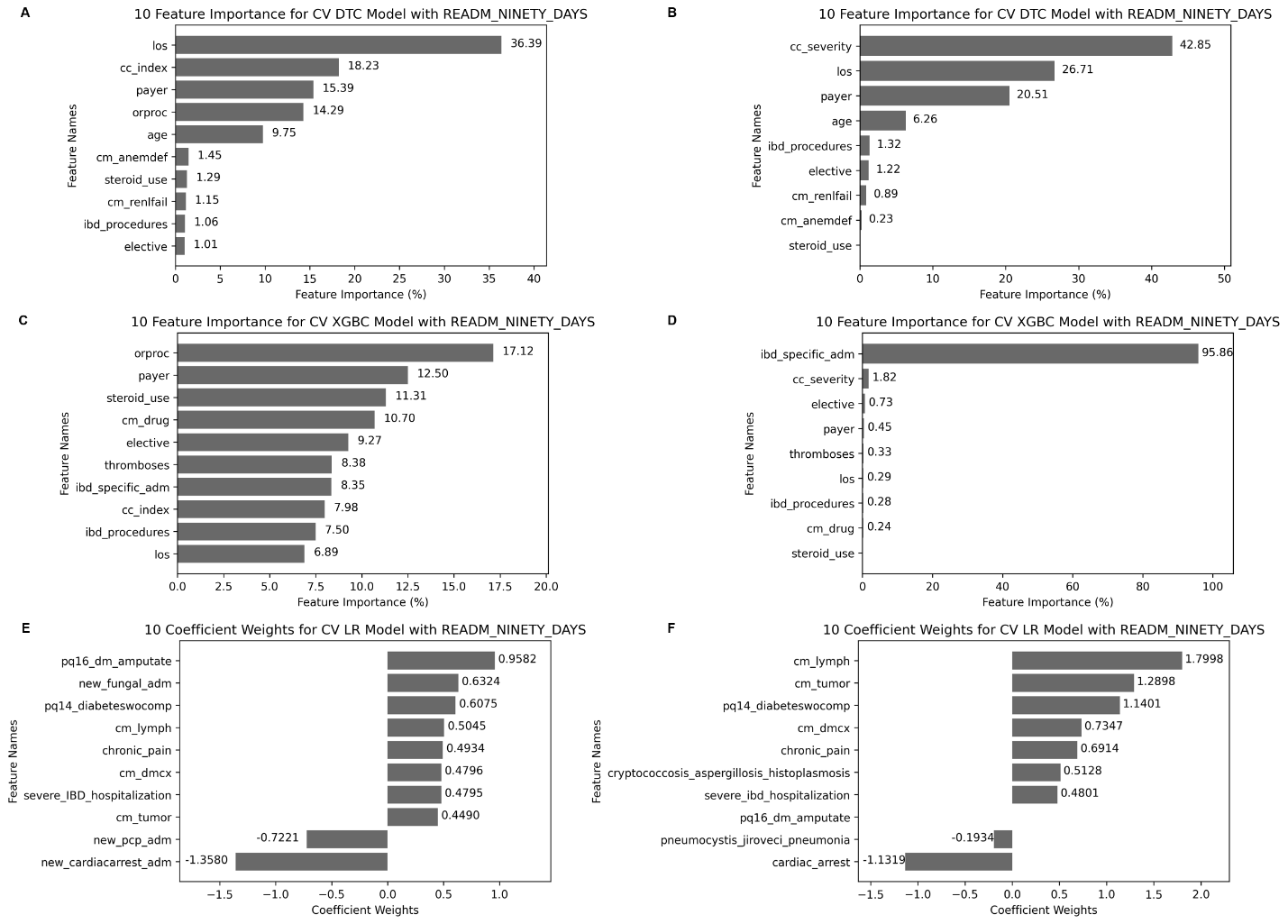


Features included into each model – variables of importance

Derivation

- **DTC:** length of stay, Charlson comorbidity index, type of insurance payer, procedure occurring in the operating room, age, medical comorbidity with deficiency anemias, corticosteroid use, medical comorbidity with renal failure, IBD-related procedures, and elective vs non-elective admission.
- **XGBoost:** procedure occurring in the operating room, types of insurance payer, corticosteroid use, medical comorbidity with drug abuse, elective vs. non-elective admission, thromboembolic events, admission for IBD-related diagnosis, Charlson comorbidity index, IBD-related procedures, and length of stay.
- **LR:** preventable admission for amputation related to diabetes, admission for fungal infection, preventable admission for diabetes without accompanying complications, medical comorbidity with lymphoma, medical comorbidity with chronic pain, complications related to diabetes, severe IBD hospitalization (defined as initial length of stay >7 days or IBD-related surgery), medical comorbidity with tumor, admission for pneumocystis jiroveci pneumonia, and admission for cardiac arrest.

Validation

- **DTC:** length of stay, Charlson comorbidity index, type of insurance payer, age, medical comorbidity with deficiency anemias, corticosteroid use, medical comorbidity with renal failure, IBD-related procedures, and elective vs non-elective admission. Of note, we could not cross-walk variable, “procedure occurring in the operating room” since it was not available in NRD 2017
- **XGBoost:** types of insurance payer, corticosteroid use, medical comorbidity with drug abuse, elective vs. non-elective admission, thromboembolic events, admission for IBD-related diagnosis, Charlson comorbidity index, IBD-related procedures, and length of stay. Of note, we could not cross-walk variable, “procedure occurring in the operating room” since it was not available in NRD 2017
- **LR:** preventable admission for amputation related to diabetes, admission for fungal infection, preventable admission for diabetes without accompanying complications, medical comorbidity with lymphoma, medical comorbidity with chronic pain, complications related to diabetes, severe IBD hospitalization (defined as initial length of stay >7 days or IBD-related surgery), medical comorbidity with tumor, admission for pneumocystis jiroveci pneumonia, and admission for cardiac arrest.
